# Foot-body coupling angle is a strong kinematic predictor of friction requirements during ladder descent: Implications for slipping risk

**DOI:** 10.1016/j.jbiomech.2025.112661

**Published:** 2025-03-28

**Authors:** Sarah C. Griffin, Violet M. Williams, Mark S. Redfern, Kurt E. Beschorner

**Affiliations:** a University of Pittsburgh, Department of Bioengineering, 301 Schenley Place, Pittsburgh, PA 15213, United States

**Keywords:** Slips, trips, and falls, Ladders, Climbing, Required coefficient of friction, Center of mass

## Abstract

Slips and falls from ladders are a common cause of workplace injuries, yet insufficient research explains the cause of these incidents. This study identifies the association between the required coefficient of friction (RCOF) and biomechanical factors (foot angle, body angle, foot-body coupling angle, foot contact velocity, foot placement, and trunk center of mass velocity) during ladder descent. In bivariate regression analyses with the ladder angle as a covariate, foot angle was negatively associated with RCOF (i.e. toe-down has a higher RCOF) (t_362_ = −19.8, p < 0.001). The body angle was negatively associated with RCOF (i.e. aligning the center of mass above the foot has a higher RCOF) (t_323_ = −3.4, p = 0.001) and there was a positive association between the foot contact velocity and the RCOF (t_359_ = 5.2, p < 0.001). Additionally, there was a strong association between the foot-body coupling angle and the RCOF (t_323_ = 21.8, p < 0.001, R^2^ = 0.606) in which the ladder angle had a small effect. A multivariate model supported that both the foot (t_322_ = −21.3, p < 0.001) and body angle (t_322_ = 8.2, p < 0.001) independently contributed to the RCOF, which supported the validity of the foot-body coupling angle. This work indicates that the foot-body coupling angle is a compelling metric associated with RCOF and could be used to train safer climbing techniques and monitor behavior to reduce ladder slip and fall events.

## 1. Introduction

Falls from ladders are a leading cause of workplace injury. In 2020, over 22,000 injuries and 160 deaths at work cited ladders as the main cause for injury (U.S. Bureau of Labor Statistics, 2023). Falls *with* a ladder can occur from the ladder slipping or tipping (Chang et al., 2005; Deschler et al., 2023; DiDomenico et al., 2013; Milligan et al., 2020; Simeonov et al., 2017; Williams et al., 2022). Falls *from* a ladder, where the climber decouples from the ladder, is an emerging area of research. One important source of falls from a ladder is slipping, which accounts for 14 % of all falls involving ladders (Cohen and Lin, 1991). Descending a ladder has been associated with a higher number of falls than ascending (Cabilan et al., 2018; Lombardi et al., 2011; Moore et al., 2009), yet slip risk during ladder descent is not well understood.

One metric to quantify slip risk is the required coefficient of friction (RCOF). It is a person- and task-specific metric that quantifies the amount of friction needed for a person to not slip while completing a task (Chang et al., 2011). RCOF has been quantified extensively for walking (Anderson et al., 2014; Beschorner et al., 2016; Chang et al., 2011; Hanson et al., 1999; Kim et al., 2005) and in a variety of tasks like turning while walking, stepping in and out of bathtubs, or stair climbing (Christina and Cavanagh, 2002; Sekiguchi et al., 2017; Siegmund et al., 2010; Yamaguchi et al., 2018; Yamaguchi et al., 2013). In walking, RCOF is positively correlated with slip events when encountering a slippery surface (Beschorner et al., 2016). A higher RCOF has been associated with increased heel contact velocity, higher walking speed, and the center of mass (COM) farther from the foot (Anderson et al., 2014; Lockhart et al., 2003; Yamaguchi and Masani, 2016; Yamaguchi et al., 2018). Understanding the relationship between biomechanical factors and RCOF can allow for training of safer techniques and identification of people at an increased slip risk.

The use of RCOF in ladder climbing is emerging and has only been described for some climbing tasks. Notably, RCOF during ladder descent has not yet been characterized. Descending has been investigated in roof-to-ladder transition and in the transition between two sections of an extension ladder (Griffin et al., 2023; Williams et al., 2023). Two biomechanical metrics have been identified as contributing to RCOF: foot angle and body angle (positioning of the trunk relative to the foot). In both steady-state ascent and descending transitions, the RCOF is significantly associated with the foot orientation, where a horizontal foot orientation is associated with a lower RCOF. The RCOF is related to the body angle during ascent (Martin et al., 2020), where the RCOF is lowest when the trunk is aligned above the foot. In the roof-to-ladder transition the medial–lateral body angle was associated with medially directed friction (Griffin et al., 2023).

Other biomechanical factors may also be relevant to RCOF during ladder climbing since they influence slip risk during climbing or RCOF during walking. Heel contact velocity and walking speed are correlated with RCOF during level walking (Anderson et al., 2014; Lockhart et al., 2003). Thus, the foot contact velocity and climbing speed for ladder climbing may also be associated with RCOF. Additionally, foot placement is significantly related to the risk of a slip and to RCOF during ascent, where the rung contacting closer to the toe is associated with a higher slip occurrence (Martin et al., 2020; Pliner et al., 2014). Therefore, foot placement is a potential predictor of the RCOF during descent.

**Fig. 1.**
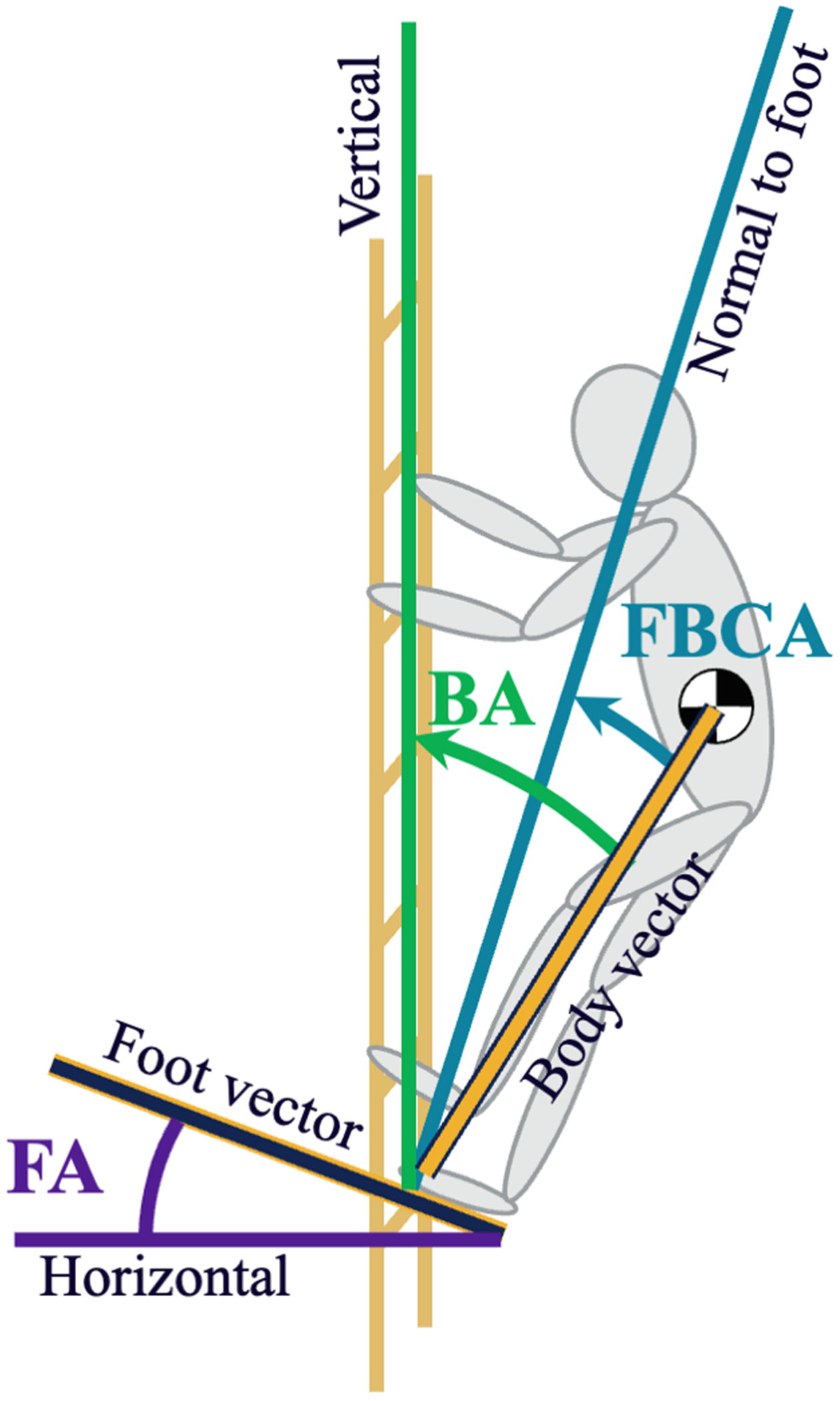
The foot angle (labeled as FA), body angle (BA) and foot-body coupling angle (labeled as FBCA) on a person climbing with a moderate foot angle and body angle (e.g. body leaned away from the ladder), and a high coupling angle.

One key difference of ladder climbing versus overground walking is that the slipping plane is defined by the foot orientation instead of the ground. Therefore, the foot orientation defines the direction of shear and normal force vectors (Griffin et al., 2023; Martin et al., 2020; Williams et al., 2023). The reaction force vector, in contrast, is influenced by the location of someone’s trunk COM relative to their contact foot (Griffin et al., 2023; Yamaguchi et al., 2013). In an ideal scenario for minimizing slip risk, the applied force would be normal to the slipping plane, resulting in no required friction. Therefore, the synchronization of foot orientation and body positioning is a potential predictor of the RCOF. In this study we propose a novel metric called the foot-body coupling angle which quantifies the relationship between the body and foot orientation by measuring the angle between the body vector (i.e. the vector from the rung contact point to the trunk COM) and the vector normal to the foot plane (Fig. 1).

The purpose of this study was to determine the relationship between the RCOF and foot and body orientation during descent of a ladder. There were two main hypotheses: 1) RCOF is negatively associated to the foot angle (i.e. a foot oriented toe-down is associated with a lower RCOF) and positively associated with the body angle (i.e. positioning the body further from the foot is associated with a higher RCOF), and 2) RCOF is positively associated with the foot-body coupling angle. In addition to these hypotheses, the impacts of foot contact velocity, climbing velocity, and foot placement on RCOF were also quantified.

## 2. Methods

### 2.1. Overview

Kinetic and kinematic data were measured while participants descended a ladder that could be set to three angles and had interchangeable rungs. This research was in accordance with the Declaration of Helsinki of 1975 and approved by the University of Pittsburgh Institutional Review Board. Written informed consent was obtained for each participant.

### 2.2. Participants

Twenty-one participants (10F, weight = 77.7 ± 17.4 kg, height = 1.69 ± 0.071 m, BMI = 26.91 ± 4.65, age = 43.2 ± 12.2) were included in this study. To be considered, they must have self-reported that they climb ladders at least ten times a year. Exclusion criteria included recent musculoskeletal injury, balance disorders, fear of heights, osteoporosis, pregnancy, back pain, or a BMI over 35. Participants had to have a weight under 136 kg to comply with safety equipment and a height under 1.96 m due to lab height restrictions.

### 2.3. Instrumentation

**Fig. 2.**
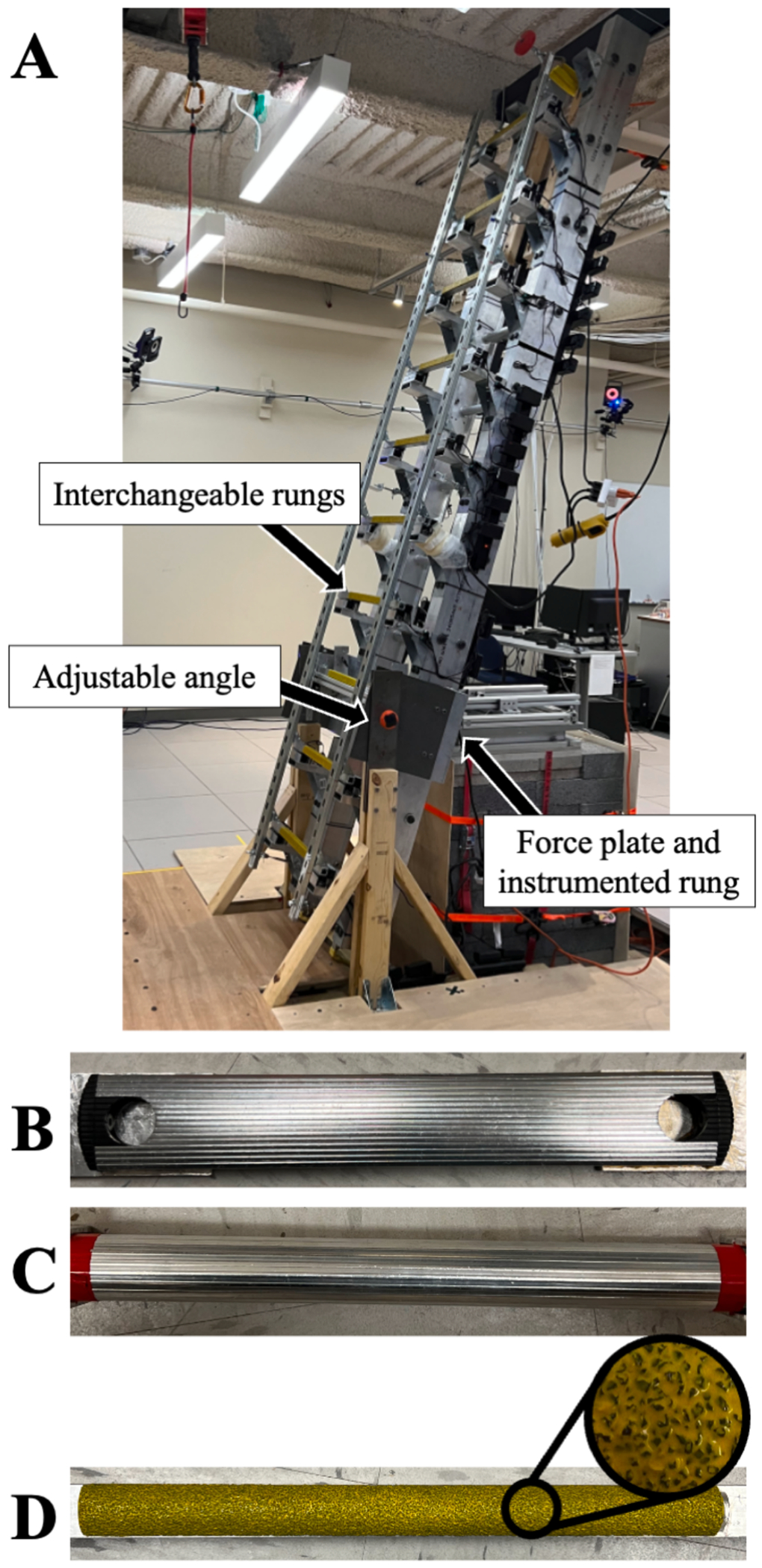
A) The testing apparatus oriented at 75 degrees. The third rung is attached to the force plate; B) A flat, wide, ridged rung (width = 50 mm); C) a round ridged rung (width = 30 mm); D) A narrow, round, rung (width = 22 mm) with a textured coating, similar to very course sandpaper, designed to provide friction in multiple directions. These rungs (B, C, D) represent a variety of designs found on commercially available ladders.

A custom instrumented ladder was created for this study (Fig. 2). The third rung of the ladder was rigidly attached to a three-dimensional force plate (AMTI Inc., Watertown, MA, USA) and separated from the rest of the ladder to allow the force plate to collect reaction forces at the shoe-rung interface. Participants were outfitted with tight fitting clothes, a safety harness, helmet, safety pads, gloves, and shoes (Vans Authentic Wide Shoe, Cost Mesa, CA). Participants donned 79 passive reflective markers (Moyer, 2006) that were tracked by motion capture cameras (Vicon Motion Systems Ltd., Centennial, CO, USA).

### 2.4. Experimental protocol

Six ladder/rung configurations were used including two ladder angles (75° and 90°) and three different rung types (Fig. 2 B–D). Multiple rung types were used in this study to ensure that the results were generalizable across rung conditions but were not part of the research questions. Separate analyses are planned to examine differences in friction requirements and slip outcomes across rung designs. Participants completed three descending trials on each ladder configuration. They were instructed to descend quickly, as though they were trying to get a task done quickly at work or at home. These instructions were to ensure that participants were moving at an urgent yet realistic pace.

### 2.5. Data Processing

The RCOF was calculated for each trial using the following methods which have been previously used for RCOF in ladder use (Griffin et al., 2023; Martin et al., 2020; Williams et al., 2023). First, kinematic data were filtered with a 7th-order lowpass Butterworth filter with a cutoff frequency of 10 Hz and kinetic data were filtered with a 9th-order lowpass Butterworth filter and cutoff frequency of 35 Hz. A vertical reference plane was created that is perpendicular to the ladder rungs and approximates the human sagittal plane. A foot vector was defined using the midpoint of markers placed on the medial and lateral heel (directly inferior to the malleoli) and the midpoint of markers placed on the anterior toes. (Griffin et al., 2023; Martin et al., 2020; Williams et al., 2023). A static trial where participants stand with level feet was used to define 0° shoe angle. The orientation of the foot vector in the vertical reference plane defined the shear contact plane (i.e. the plane tangent to the rung’s contact surface along which a slip would occur). The reaction forces from the force plate were rotated into this plane. Time-series friction to normal force ratio was generated for the duration of foot contact using the resultant shear force (i.e. AP and ML shear combined) from the rotated coordinate system (see Supplementary Materials).

**Fig. 3.**
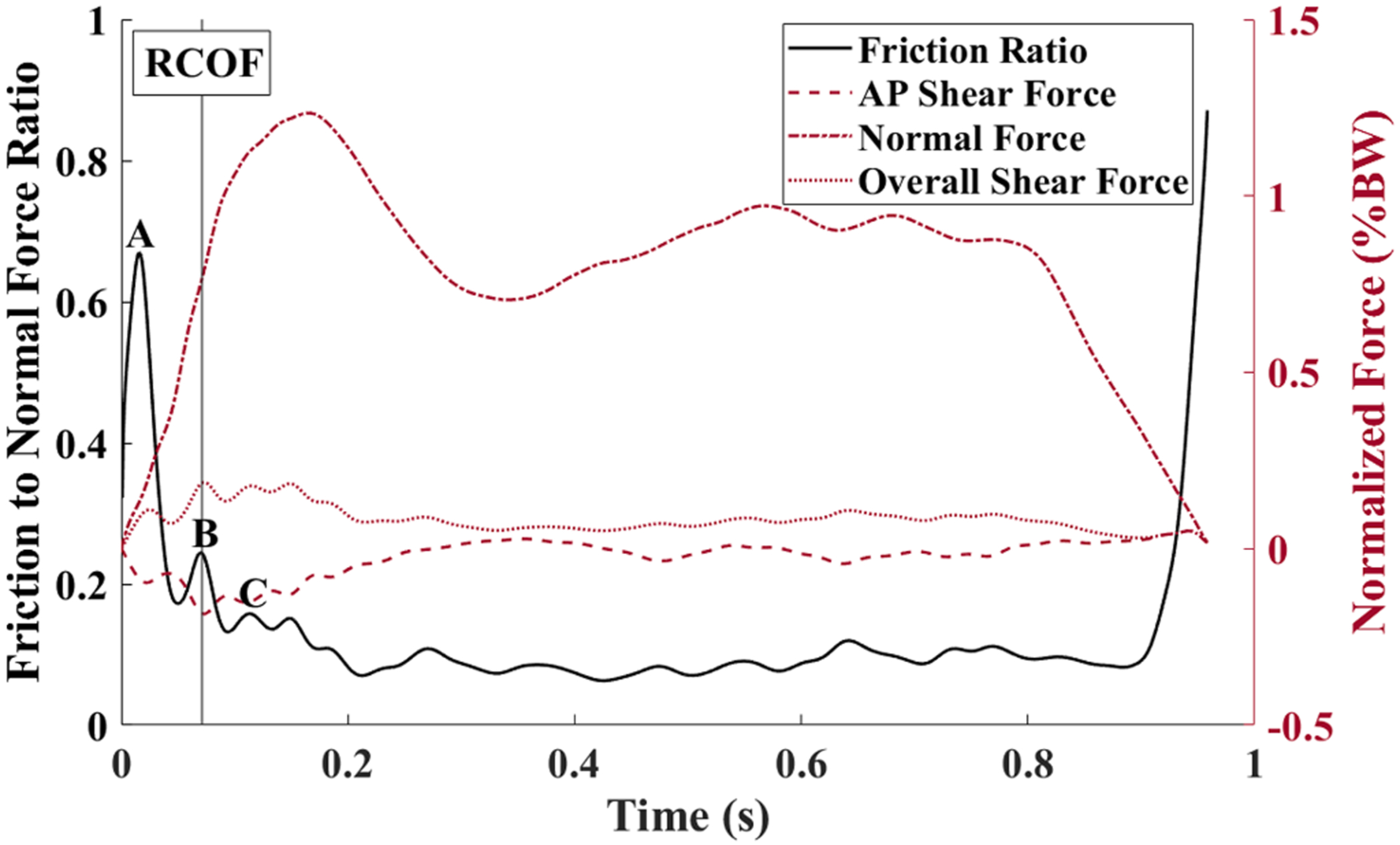
Time-series plot of a typical friction to normal force ratio and the corresponding forces. The friction to normal force ratio is plotted in black on the left axes and the forces normalized to body weight (BW) are plotted in maroon on the right axes. The peaks labeled by the cluster analysis are denoted as A, B, or C. The time of RCOF is denoted with a vertical line. The negatively directed AP shear force at time of RCOF indicates a tendency for a forward slip.

The RCOF peak was selected using cluster analysis methodology (Williams et al., 2023). Using this method to select similar peaks across trials limits the user bias associated with manually selecting RCOF peaks. The first three local maxima from each trial were found and input into the clustering algorithm. Data points were sorted by the value of the local maximum, its corresponding normal force (% body weight), and the time after foot contact (% stance). The algorithm generated three clusters (A, B, C; Fig. 3). The first group (A) represented the initial, high value peak that corresponds to low normal forces during loading. This peak was excluded because the low forces are insufficient to initiate a slip (Griffin et al., 2023; Martin et al., 2020; Williams et al., 2023). The second (B) and third (C) groups together captured the peaks which may be relevant for the RCOF. The maximum value that was classified as a peak B or C group was chosen as the RCOF for each trial (Williams et al., 2023).

Five kinematic metrics were calculated at the time of RCOF: 1) foot angle, 2) body angle, 3) foot-body coupling angle, 4) foot placement and 5) trunk COM velocity. The sixth metric, foot contact velocity, was calculated at the time of foot contact as measured by when force was first detected by the force plate (threshold = 25 N). The foot angle was in the vertical reference plane, which was the plane perpendicular to the shear contact plane and the horizontal plane. For the body angle, the angle was calculated between vertical and a vector from the anterior toe (defined as the midpoint between markers placed on the distal ends of the first and fifth phalanges) to the trunk COM (Griffin et al., 2023; Martin et al., 2020; Pliner et al., 2014). The foot-body coupling angle was the angle between the body vector and a vector perpendicular to the foot. This was calculated at the time of RCOF and was equivalent to subtracting the foot angle from the body angle. A value of zero indicates perfect synchronization of foot and body angle which was expected to minimize required friction. The trunk COM velocity was calculated by determining the magnitude of the first derivative of the position of the trunk COM. Foot position was found by determining the distance between the rung and the anterior toe in the anterior-posterior direction (Pliner et al., 2014). This value was normalized to foot length. To determine foot contact velocity, the midpoint between a marker located on the medial surface of the first metatarsal and a marker located on the lateral surface of the fifth metatarsal was calculated. The first time derivate of the position of this point projected into the shear contact plane was used as the foot contact velocity.

### 2.6. Statistical analysis

Six linear regression models were used to determine relationships between RCOF and the independent variables: foot angle, body angle, foot-body coupling angle, foot contact velocity, trunk COM velocity, and foot position. All models included ladder angle as a covariate due to its relationship with both kinetics and kinematics (Lee et al., 1994; Martin et al., 2020). Models were evaluated for overall significance using ANOVA tests and individual regressors were evaluated with t-tests.

Multivariate methods were used to test the underlying rationale for foot-body coupling angle. This metric suggests that the coordination between the foot and body angle is more important than either in isolation. If this were true, then both foot angle and body angle should independently contribute to RCOF in a multivariate model. Further-more, these methods could determine if ladder angle influenced RCOF after controlling for foot and body angle. Thus, a multivariate model was considered with foot angle, body angle, and ladder angle as regressors and RCOF as the dependent variable. The model’s significance was evaluated with an F-test and the individual independent variables were evaluated with t-tests. Statistical analyses were completed in JMP 17 Pro (JMP^®^, Version 17 Pro. SAS Institute Inc., Cary, NC, 1989–2024) with a significance level of 0.05.

## 3. Results

The average RCOF was 0.25 with a standard deviation of 0.085. The direction of the anterior/posterior frictional forces, on average, suggests the risk of a forward slip (Fig. 3). No slips occurred during the baseline trials that are reported in this study. Central tendency and spread for the considered biomechanical variables are presented in Table 1.

**Fig. 4.**
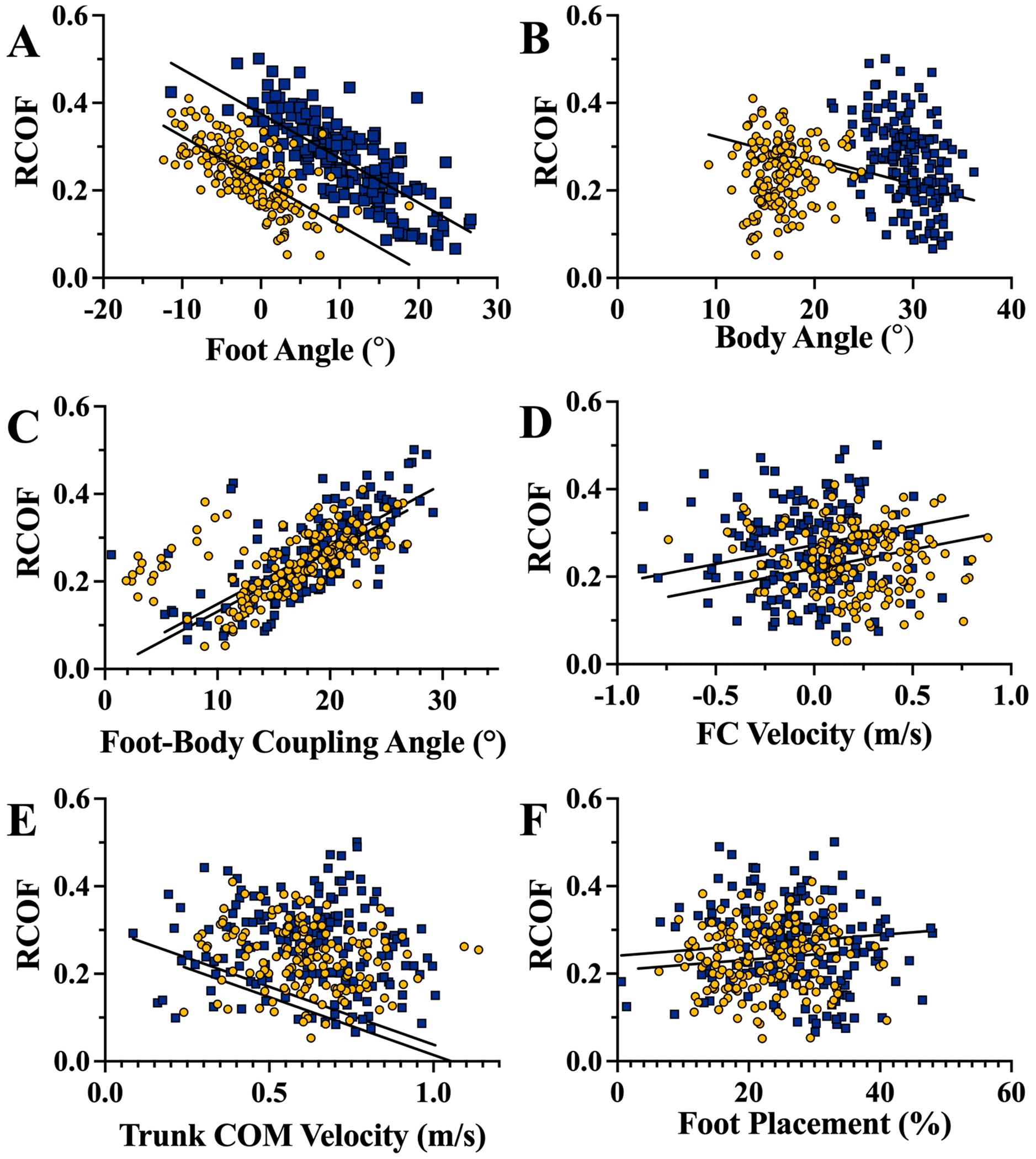
Regression plots for the individual biomechanical factors (A: foot angle, B: body angle, C: foot-body coupling angle, D: foot contact velocity, E: trunk COM velocity, and F: foot placement) against the RCOF with ladder angle as a covariate. All associations were significant, but the effect size was small for the trunk COM velocity and foot placement. Yellow circular markers were collected on the 75° ladder angle and blue square markers were collected on the 90° ladder. (For interpretation of the references to color in this figure legend, the reader is referred to the web version of this article.)

The RCOF had a strong, positive association with the foot-body coupling angle (t_323_ = 21.8, p < 0.001, R^2^ = 0.606). Also, the foot angle had a strong negative association with the RCOF (t_362_ = −19.8, p < 0.001, R^2^ = 0.534). The body angle had a weak, positive association with the RCOF (t_323_ = −3.4, p = 0.001, R^2^ = 0.061). Finally, weak, negative associations were found between the RCOF and the foot contact velocity (t_359_ = 5.2, p < 0.001, R^2^ = 0.097), the RCOF and trunk COM velocity (t_321_ = −2.4, p = 0.015, R^2^ = 0.047), and the RCOF and foot placement location (t_347_ = 2.2, p = 0.030, R^2^ = 0.043) (Fig. 4). Ladder angle was significant in all models. The 90° angle was generally associated with a higher RCOF than the 75° angle. The size of this effect (the difference between the least squares mean RCOF for 90° and 75°) was 0.153 for foot angle, 0.105 for body angle, 0.017 for foot-body coupling angle, 0.054 for foot contact velocity, 0.024 for trunk COM velocity, and 0.034 for foot placement.

**Fig. 5.**
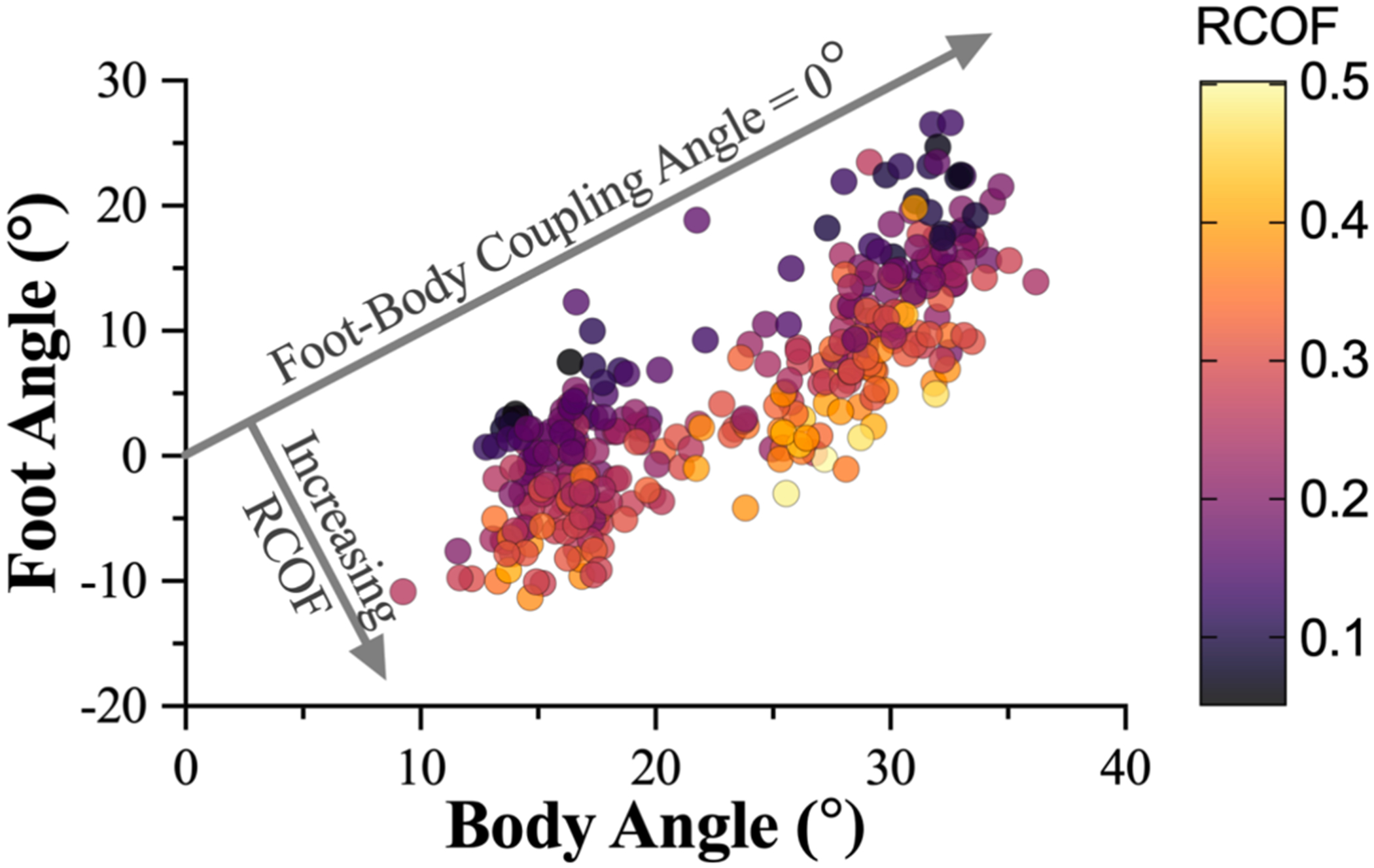
The impact of the foot and body angle on the RCOF while descending a ladder. The RCOF is indicated by marker color using a heat map where a lighter yellow color is a high RCOF and a dark purple color is a low RCOF. The graph is annotated to show the line where foot-body coupling angle is zero and RCOF is expected to be lowest. (For interpretation of the references to color in this figure legend, the reader is referred to the web version of this article.)

The multivariate model (F_3,322_ = 168.0, p < 0.001, R^2^ = 0.610, Fig. 5) and all of the factors (foot angle (t_322_ = −21.31, p < 0.001), body angle (t_322_ = 8.17, p < 0.001), and ladder angle (t_322_ = −2.87p = 0.004)) were significant predictors of RCOF. The effect of ladder angle in this model was small. The significance of both the foot angle and body angle indicates that they provide unique information and explains the high correlation observed for the foot-body coupling angle (Fig. 4C). In this model, foot orientation was negatively correlated to RCOF where the lowest RCOF was found when the foot was in dorsiflexion (toe-up). A 10° increase in the foot angle was associated with a decrease of 0.135 in RCOF. The body angle was positively associated with RCOF where a 10° increase in the body angle was associated with an increase of 0.113 in the RCOF. Thus, each angle had a similar contribution to RCOF further supporting combining them to a single metric.

## 4. Discussion

As predicted, all metrics (foot angle, body angle, foot-body coupling angle, foot contact velocity, trunk COM velocity, and foot placement) were significantly associated with RCOF. The effects for the models with body angle, trunk COM velocity, foot placement, and foot contact velocity were small. However, the effect of foot angle and foot-body coupling angle was large. The foot-body coupling angle had an advantage over the foot angle since it better generalized across the two ladder angles. In the multivariate model, both foot and body angle were significantly associated with RCOF indicating that both variables (and thus the foot-body coupling angle) provide unique contributions to predict the RCOF.

The results indicate that the foot-body coupling angle is a promising predictor for RCOF (during ladder climbing). When used as the sole regressor for RCOF (i.e. a model that excludes the ladder angle), the model had an R^2^ value equal to 0.596, indicating that the foot-body coupling angle can explain a majority of the variance in RCOF. Theoretically, it follows that an ideal climbing position (where the foot-body coupling angle is 0°) occurs when the body vector is perpendicular to the foot vector. Another promising aspect of the coupling angle is that the ladder angle has a small effect on the coupling angle’s relationship with the RCOF. This small effect in both the controlled bivariate analysis and the multivariate test indicates that the foot-body coupling angle is robust to changes in conditions. This work indicates that while the individual metrics do provide meaningful information, the foot-body coupling angle is the best individual predictor of the RCOF.

In level walking, the RCOF is highly correlated (R = 0.94) to the tangent of the angle formed by a vector traveling from the center of pressure (COP) to the whole-body COM (Yamaguchi and Masani, 2016). This can be explained mathematically by a two-legged inverted pendulum model. In ladder climbing, the foot-body coupling is more appropriate than the COP-COM angle because it also considers the shear contact plane orientation. In a post-hoc analysis, we determined that the tangent of the foot-body coupling angle was correlated with the RCOF (R = 0.77), but this correlation was not as strong as that reported in walking (R = 0.94) (Yamaguchi and Masani, 2016). Typically, the strong correlation between the COM angle and force angle is because conservation of angular momentum requires the ground reaction force to be oriented toward the COM to minimize angular acceleration, especially during single stance. However, the arms act as a support during ladder climbing, which may enable the ground reaction forces to deviate more from the body’s COM without inducing angular acceleration. Future work can develop a mechanical model which considers the upper limbs in ladder climbing.

Foot angle was negatively associated with RCOF in both its bivariate correlation model and the multivariate model, meaning that orienting the foot toe-up in space (dorsiflexing) decreases RCOF. This is consistent to what was observed in a roof-to-ladder transition and while descending an extension ladder (Griffin et al., 2023; Williams et al., 2023). However, the opposite relationship was present in ladder ascent, where the lowest RCOF was captured when someone had their foot in plantarflexion (Martin et al., 2020; Pliner et al., 2014). The difference may be due to the fundamental differences in the movements associated with climbing up and down a ladder.

There are some limitations within this study that should be noted. Due to the height restrictions of the laboratory, the participants could only descend from the fifth rung of the ladder, meaning that the maximum height of their feet was around 1.53 m high, which is lower than what is typically seen on a worksite. The perceived risk of climbing to a lower height may have altered the participants’ behavior (Simeonov et al., 2003). Further, the safety equipment (i.e. the shin guards, elbow pads, and knee pads) may have altered the way that the participants were able to move, or they may have altered their behavior due to being observed by the researchers. Finally, more perturbation studies are needed to confirm the relationship between RCOF and slipping risk for ladder climbing.

The significance of the foot-body coupling angle could provide an opportunity for biomechanical training to reduce slip risk. Future work can identify a meaningful way to cue workers to lower their foot-body coupling angle, potentially through biofeedback or training on proper technique. Additionally, this metric could be used to monitor the safety of ladder climbing. With the expansion of safety monitoring systems including camera- and wearable-based technology, foot-body coupling angle could be targeted to identify individuals and work tasks associated with an elevated risk of slip. Finally, minimizing the foot-body coupling angle offers unique benefits for reducing slip risk. Individuals of different sizes or strengths may not be able to climb with a specific body angle. However, many different combinations of foot and body positionings can minimize the slip risk and a person can customize their strategy to their climbing style (climbers who prefer to climb with a large body angle can adapt their foot angle and vice versa). Thus, this is a potentially more flexible intervention than training climbing technique based on foot or trunk angle alone.

This study found that the foot-body coupling angle is a strong predictor of RCOF while descending a ladder. A person who synchronizes their trunk COM location and their foot orientation to minimize their foot-body coupling angle while climbing down a ladder will reduce their slipping risk. These results indicate that there may be an opportunity to train safer ladder climbing techniques to reduce the frequency of ladder slip and fall events.

## Supplementary Material

1

## Figures and Tables

**Table 1:** Descriptive statistics for the biomechanical factors. All factors except the foot contact velocity are calculated at the time of the RCOF. Foot contact velocity was calculated at the time of foot contact.

Metric (Unit)	Mean	Standard deviation
Foot angle (°)	4.5	8.5
Body angle (°)	23.2	7.0
Foot-body coupling angle (°)	18.1	4.8
Foot contact velocity (m/s)	0.08	0.31
Trunk COM velocity (m/s)	0.62	0.18
Foot position (% foot length)	24.6	8.4

## Appendix A. Supplementary material

Supplementary data to this article can be found online at https://doi.org/10.1016/j.jbiomech.2025.112661.
