# Supplementary material for: Foot-body coupling angle is a strong kinematic predictor of friction requirements during ladder descent: Implications for slipping risk
Source: J Biomech. Author manuscript; Available in PMC 2026 May 22. (PMC13195415; doi:10.1016/j.jbiomech.2025.112661)
Supplement: 1 [file NIHMS2173336-supplement-1.docx]

**Appendix**

The RCOF peak is extracted from a time series ratio of shear to normal forces during the time that the foot is contacting the instrumented rung. The shear and normal forces are calculated with respect to the shear contact plane, defined by the foot orientation. Equations 1 and 2 are used to calculate the time series signal.

Eq. 1 $\left[ \begin{matrix} F_{x, Foot} \\ F_{y, Foot} \\ F_{z,Foot} \end{matrix} \right]=\left[ \begin{matrix} -1 & 0 & 0 \\ 0 & -\cos\left( \alpha\right) & \sin\left( \alpha\right) \\ 0 & \sin\left( \alpha\right) & \cos\left( \alpha\right) \end{matrix} \right]$*$\left[ \begin{matrix} F_{x,FP} \\ F_{y,FP} \\ F_{z,FP} \end{matrix} \right]$

Eq. 2 $\frac{F_{Friction}}{F_{Normal}}(t)= \frac{\sqrt{F_{x,Foot}^{2}+F_{y,Foot}^{2}}}{F_{z,Foot}}$

Where F_FP_ represents the three-dimensional forces measured by the force plate, 𝛼 represents the foot angle in the vertical reference plane (perpendicular to the ladder rungs), and F_Foot_ values are the transformed forces in the foot coordinate system used to calculate RCOF. The RCOF is a peak chosen from the time-series friction to normal force ratio ($\frac{F_{Friction}}{F_{Normal}}$).
